# Defining the Functional Targets of Cap‘n’collar Transcription Factors NRF1, NRF2, and NRF3

**DOI:** 10.3390/antiox9101025

**Published:** 2020-10-21

**Authors:** Lara Ibrahim, Jaleh Mesgarzadeh, Ian Xu, Evan T. Powers, R. Luke Wiseman, Michael J. Bollong

**Affiliations:** 1 Department of Molecular Medicine, The Scripps Research Institute, 10550 North Torrey Pines Road, La Jolla, CA 92037, USA; librahim@scripps.edu (L.I.); jmesgarz@scripps.edu (J.M.); ijkxuluo7000@gmail.com (I.X.); wiseman@scripps.edu (R.L.W.); 2 Department of Chemistry, The Scripps Research Institute, 10550 North Torrey Pines Road, La Jolla, CA 92037, USA; epowers@scripps.edu

**Keywords:** oxidative stress response, NRF transcription factors, transcriptomics, proteomics, cellular pathway analysis

## Abstract

The NRF transcription factors NRF1, NRF2, and NRF3, are a subset of Cap‘n’collar transcriptional regulators which modulate the expression of genes harboring antioxidant-response element (ARE) sequences within their genomic loci. Despite the emerging physiological importance of NRF family members, the repertoire of their genetic targets remains incompletely defined. Here we use RNA-sequencing-based transcriptional profiling and quantitative proteomics to delineate the overlapping and differential genetic programs effected by the three NRF transcription factors. We then create consensus target gene sets regulated by NRF1, NRF2, and NRF3 and define the integrity of these gene sets for probing NRF activity in mammalian cell culture and human tissues. Together, our data provide a quantitative assessment of how NRF family members sculpt proteomes and transcriptomes, providing a framework to understand the critical physiological importance of NRF transcription factors and to establish pharmacologic approaches for therapeutically activating these transcriptional programs in disease.

## 1. Introduction

The nuclear factor (erythroid 2)-like (NRF) transcription factors are a family of basic leucine zipper (bZIP)-containing transcriptional regulators related to nuclear factor erythroid 2 (NFE2, also called p45), that are characterized by a conserved 43 amino acid Cap‘n’collar (CNC) motif [1]. Vertebrate NRF transcription family members consist of three closely related factors, NRF1 (NFE2L1), NRF2 (NFE2L2), and NRF3 (NFE2L3) [1]. In the nucleus, NRF transcription factors dimerize with small MAF proteins (small musculoaponeurotic fibrosarcoma; MAFF, MAFG, and MAFK) to modulate the transcription of genes containing antioxidant response elements (AREs; consensus sequence = TGABNNGC) within their genomic loci [1].

Unlike NFE2, which plays a developmental role in promoting megakaryopoiesis, the NFE2-like NRF transcription factors inducibly respond to the presence of cellular stressors [2]. A seminal contribution supporting this hypothesis was the early association of NRF2 to the inducible transcriptional upregulation of genes containing ARE sites within their promoters [3]. A related effort determined that NRF2 knockout animals while displaying no overt developmental defects, are prone to carcinogenesis, and are critically sensitive to oxidative stress [4]. NRF2 is now appreciated as the master regulator of oxidative stress resistance in mammalian cells. As such, small molecules that activate NRF2 have and continue to be developed for indications etiologically defined by oxidative stress, such as chronic kidney disease [5,6]. Further, deregulated NRF2 activity has been associated with various disease states such as cancer [7], neurodegeneration [8], inflammation [9], and diabetes [10]. 

Unlike NRF2, the functional importance of other NRF transcription factors is less well defined. An initial focus of NRF1 research relied on the early observation that NRF1 deficient animals die late during embryonic development due to insufficient hepatic erythropoiesis [1]. More recently, a renewed interest in NRF1 was precipitated by seminal work by Deshaies and colleagues demonstrating that NRF1 mediates the proteasomal transcriptional “bounce back” in response to proteasome inhibitor treatment [11]. In contrast to NRF2, NRF1 is localized to the ER and must be actively transported across the ER membrane, deglycosylated, and proteolytically cleaved to release a transcriptionally active and nuclear-localized form of the transcription factor [12]. In this way, the rate of proteasome-based degradation of newly transported NRF1 in the cytoplasm serves as an adjustable rheostat for NRF1-driven cellular proteasomal activity. Despite the mounting evidence for an essential role of NRF1 in physiology, the full spectrum of its transcriptional repertoire has yet to be fully defined.

The least explored of the NRF transcription factor family is NRF3, likely stemming from the observation that knockout animals display no obvious phenotypes and do not additively contribute to the deleterious effects of animals lacking NRF2 or p45 [1]. Recently, a number of reports have demonstrated an essentiality for NRF3 in various cancer cell types, but the role of NRF3 in normal physiology remains largely enigmatic [13,14].

The evolutionary emergence of three closely related NRF family members in vertebrates is thought to have arisen from genomic duplications, as NRF1, NRF2, NRF3, and NFE2 all reside near related homeobox genes (i.e., HOXB, HOXD, HOXA, and HOXC, respectively) [15]. In the lower invertebrates however, only one CNC-containing transcription factor is present. For example, *C. elegans* possess only the CNC-containing gene SKN-1 (Skinhead family member 1) whereas flies contain only the CNC-containing factor CncC [16,17]. It is thought that these CNC transcription factors enact broad stress-related transcriptional programs to combat several cellular stressors such as oxidative injury and protein aggregation, effectively combining the known transcriptional programs of metazoan NRF1 and NRF2. While recent research has spelled out important roles for NRF1 and NRF2 in regulating cellular physiology, the functional importance for three stress-inducible CNC-containing transcription factors with overlapping, but distinct, transcriptional profiles in vertebrates is not completely defined. One approach to deconvolute the diverse functional roles for these transcription factors is to define transcriptional gene sets that can be used to define transcription factor activity both in cell culture and mammalian tissues [18]. To date, only one recent study by Liu et al. has aimed to comparatively delineate the transcriptional programs enacted by NRF factors [19]. Here, we build upon this work using RNA-sequencing and quantitative proteomics to define the unique and overlapping programs enacted by individual NRF family members, providing a framework to deconvolute the integrated signaling of these transcription factors in the context of health and disease.

## 2. Materials and Methods

### 2.1. Cell Culture

HEK293T cells were from American Type Culture Collection (ATCC, CRL-11268). Cells were maintained in DMEM medium (Corning, Corning, NY, USA) and supplemented with 10% FBS (fetal bovine serum, Gibco, Waltham, MA, USA) and 1% Penicillin-Streptomycin (Gibco).

### 2.2. Cloning

The FLAG-NRF2 expression construct was obtained from Addgene (NC16 pCDNA3.1 FLAG NRF2, Plasmid #36971, NM_006164.5), and a site-directed mutagenesis kit (Q5 Site-Directed Mutagenesis Kit, NEB, Ipswich, MA, USA) was used to introduce the T80D mutation. Codon-optimized sequences encoding truncated, FLAG-tagged transgenes of NRF1 (NM_003204.3) and NRF3 (NM_004289.7) were obtained from Integrated DNA Technologies as gBlock HiFi Gene Fragments and cloned into the pCDNA3.1 backbone from FLAG-NRF2 via Gibson assembly using a HiFi DNA Assembly Cloning Kit (NEB).

### 2.3. Immunoblotting

Plasmids were transiently transfected for transgene expression in HEK293T cells using FuGENE (4 µL FuGENE per 1 µg of DNA) in 100 μL of Opti-MEM (Gibco) per well of a six-well plate (2 μg DNA per well). After 24 h of expression, cells were lysed with 1× RIPA buffer (Millipore, Burlington, MA, USA). Samples were prepared for SDS–PAGE by heating to 95 °C for 5 min, cooled to room temperature, resolved on NuPAGE Novex 4–12% Bis-Tris Protein Gels (Invitrogen, Waltham, MA, USA), and then transferred to PVDF membranes (Bio-Rad Laboratories, Hercules, CA, USA) using a semidry transfer apparatus (Bio-Rad Laboratories). Membranes were blocked with 5% non-fat dry milk (Bio-Rad Laboratories) in Tris-buffered saline (TBS, Corning) containing 0.1% Tween-20 (TBST, Corning) and probed with primary (overnight at 4 °C) and secondary (1 h at room temperature) antibodies in blocking buffer. Primary antibodies used in this study were anti-FLAG-M2 (1:1000, F1804, Sigma Aldrich, St. Louis, MO, USA) and TUBG1 (1:1000; 5886, Cell Signaling Technologies, Danvers, MA, USA). A secondary HRP-conjugated rat anti-mouse antibody (Sigma Aldrich) was used at 1:10,000 dilution in 5% milk in TBST and incubated for 1 h before washing for an additional hour. Luminescent signal was recording using autoradiography film (Life Technologies, Waltham, MA, USA) after incubation with SuperSignal West Dura substrate (Life Technologies).

### 2.4. Quantitative Reverse Transcription PCR (qRT-PCR)

After 24 h of transgene expression as above, cells were collected by trypsinization and subsequent centrifugation at 1200× *g*. RNA was isolated using an RNeasy kit (Qiagen, Hilden, Germany) and RNA concentrations measured using a NanoDrop instrument. 1 ng of RNA was then subjected to reverse transcription reaction with oligo dT primers using a SuperScript III First-Strand Synthesis kit from Invitrogen. Quantitative RT-PCR reactions were measured on a Viia 7 Real-Time PCR system (Thermo) using a TB Green master mix from Takara. Primers used were *HMOX1* (forward: GAGTGTAAGGACCCATCGGA, reverse: GCCAGCAACAAAGTGCAAG) and *NQO1* (forward: GCCTCCTTCATGGCATAGTT, reverse: GGACTGCACCAGAGCCAT). Reactions were normalized to *TUBG1* levels (forward: ATCTGCCTCCCGGTCTATG, reverse: TACCTGTCGGAACATGGAGG) and relative transcript abundance calculated using the comparative *C_t_* method.

### 2.5. Luciferase Reporter Assays

5 × 10^3^ HEK293T cells were plated per well in white 384-well plates in 40 μL of growth medium and transfected with 50 ng of the ARE-LUC reporter plasmid and 50 ng of the indicated FLAG-NRF construct in 10 uL of Opti-MEM using PEI (25 kDa polyethyleneimine (Polysciences, Warrington, PA, USA); 1 µL of PEI to 1 µg of DNA). After 24 h incubation, luminescence values were recorded on an Envision instrument (Perkin Elmer) after the addition of 30 μL of Bright Glo reagent solution (Promega, diluted 1:3 in water).

### 2.6. RNA Sequencing (RNA-Seq) Experiments

Total RNA was extracted from HEK293T cells after 24 h of transient transgene expression using an RNeasy kit (Qiagen). RNA sequencing was performed at BGI using the DNAseq Technology platform. RNA sequencing data have been deposited in the NCBI Gene Expression Omnibus and are accessible through GEO Series accession number GSE159230. Transcript abundance was estimated using DNASTAR’s Lasergene Genomics Suite and the statistical significance of differentially expressed transcripts was determined by two-sample unequal variance *t*-tests. Gene set enrichment analysis (GSEA, Broad Institute) was performed using the Java application, and results replotted using R (https://www.r-project.org/).

### 2.7. Mass Spectrometry-Based Quantitative Proteomics Experiments

After 24 h of transgene expression, HEK293T cells were lysed with 1 × RIPA buffer and subjected to TMT labeling and mass spectrometry as previously described [20]. In brief, the protein was precipitated in chloroform and methanol, then dissolved in Rapigest (Waters, Milford, MA, USA). Disulfide bonds were reduced using TCEP (tris(2-carboxyethyl)phosphine) (Thermo Fisher, Waltham, MA, USA) in HEPES (4-(2-hydroxyethyl)-1-piperazineethanesulfonic acid) (Thermo Fisher)-buffered solution, followed by S-alkylation with chloroacetamide (Thermo Scientific). Samples were then enzymatically digested with Porcine Trypsin (Promega, Madison, WI, USA) overnight and then labeled using 11-plex Tandem Mass Tags (TMT) Isobaric Mass Tag labeling reagents (Thermo Fisher). TMT labeled samples were then combined and fractionated using the Pierce High pH Reversed-Phase Peptide Fractionation Kit (Thermo Scientific). Fractionated samples were then injected onto a C18 analytical column and subjected to MS1 and MS2 fragmentation on a Thermo Scientific Q Exactive HF Orbitrap. Protein identification and quantification were performed using the Integrated Proteomics Pipeline (IP2) [21,22]. Tandem mass spectra in the form of MS1 and MS2 files were searched against the current reviewed UniProt human protein database.

## 3. Results

### 3.1. Design and Validation of an NRF Transgene Overexpression Platform

**Figure 1:**
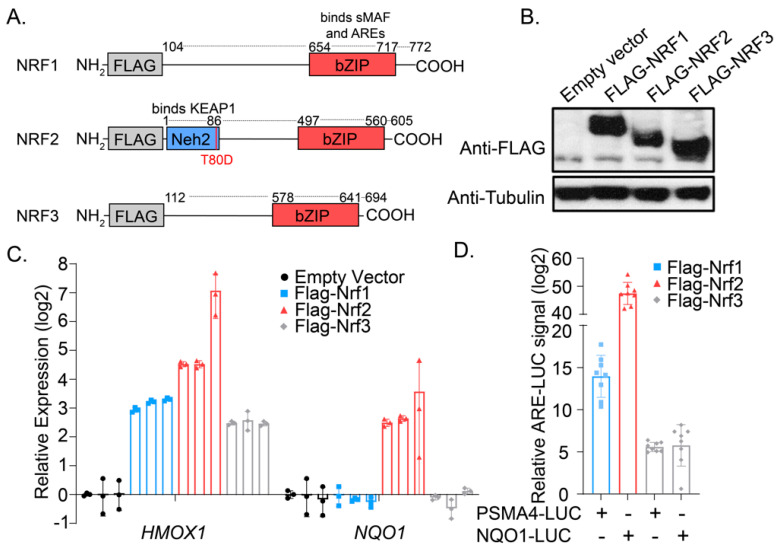
An overexpression system for evaluating the activities of NRF transcription factors in HEK293T cells. (**A**) Schematic depicting the FLAG-NRF constructs used in this work; numbers above each transgene indicate the amino acid positions of the reference ORF. (**B**) Western blotting analysis for FLAG protein content from HEK293T cells 24 h after transfection with the indicated constructs. (**C**) Transcript levels of *HMOX1* and *NQO1* as measured by qRT-PCR from HEK293T cells 24 h after transfection with the indicated constructs (*n =* 3; mean and s.d.). (**D**) Relative ARE reporter activity from HEK293T cells overexpressing the indicated FLAG-NRF construct (*n =* 8; mean and s.d.).

We envisioned a cellular platform in which transient overexpression of constitutively active versions of NRF transgenes might allow for interrogation of their impact on the cellular transcriptome and proteome. As such, we first designed overexpression constructs wherein the encoded NRF transgenes would be minimally influenced by cellular regulatory machinery. As NRF1 and NRF3 are anchored N-terminally within the ER lumen, we constructed expression plasmids lacking the N-terminal ER-targeting sequence, a modification that allows for cytoplasmic translation and thus bypass of the deglycosylation and cleavage steps required for exit from the ER [13,23]. Unique among this family, NRF2 is cytoplasmically localized where it is continually sent for proteasomal degradation through its interactions with the repressor and oxidative-stress sensing protein KEAP1 [24]. We introduced a mutation (T80D) in the Neh1 domain of NRF2, which has been reported to inhibit its interactions with KEAP1 and to promote constitutive transcriptional activation [25]. All transgenes were N-terminally FLAG-tagged to allow for comparative evaluation of protein expression levels and to minimally interfere with the C-terminal CNC-bZIP domain, which dimerizes with small MAFs and binds DNA (Figure 1A). We next identified optimized transfection and expression conditions using HEK293T cells, confirming similar levels of NRF transgenes by anti-FLAG Western blotting (Figure 1B). Consistent with reports suggesting the antioxidant gene Heme Oxygenase 1 (*HMOX1*) as a promiscuous NRF target gene, expression of NRF1, NRF2, or NRF3 induced significant expression of *HMOX1* relative to vector controls as evaluated by qRT-PCR from HEK293T cells, suggesting the designed transgenes are transcriptionally active (Figure 1C). Further, we found that overexpression of NRF2, but not NRF1 or NRF3, induced expression of the NRF2-selective transcriptional target *NQO1* (NAD(P)H dehydrogenase [quinone] 1) by RT-qPCR, confirming that the designed transgenes are capable of upregulating specific target transcripts. This selectivity was further demonstrated by showing overexpression of NRF1 and NRF2 in HEK293T cells promoted robust activation of co-transfected luciferase reporters PSMA4-ARE-LUC and NQO1-ARE-LUC, respectively (Figure 1D). These reporter plasmids harbor the ARE sequences from Proteasome Subunit Alpha Type-4 (*PSMA4*) and *NQO1*, which we previously demonstrated to specifically capture pharmacological activation of NRF1 and NRF2 activation in cells, respectively [26]. NRF3 overexpression promoted only a mild induction of either reporter, suggesting these plasmids were largely orthogonal reporters of NRF activity and that the NRF expression constructs in this work were sufficiently active to induce robust and distinct transcriptional programs in cells. 

### 3.2. Defining Transcriptomic and Proteomic Targets of NRF Family Members

**Figure 2:**
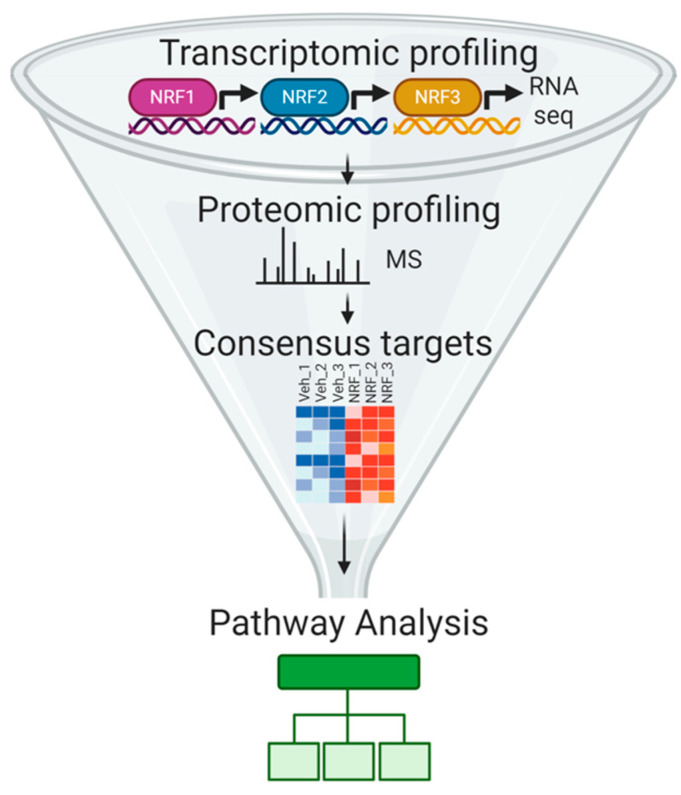
Schematic depicting the strategy used in this work to define the functional targets of the NRF transcription factors.

To identify the transcripts, protein levels, and genetic programs regulated by NRF overexpression, we coupled our overexpression platform to a data acquisition and analysis pipeline consisting of RNA-sequencing in conjunction with mass spectrometry-based quantitative proteomics (Figure 2; Supplemental Figures S1–S3). This approach allowed for the detection of differentially expressed transcripts and proteins in HEK293T cells overexpressing the indicated NRF transgenes. The targets of NRF members were verified at the transcript and protein level by statistical significance testing and were then analyzed by pathway association programs such as Gene Set Enrichment Analysis (GSEA) [27,28] and Database for Annotation, Visualization and Integrated Discovery (DAVID) [29], as discussed below.

#### 3.2.1. RNA Sequencing-Based Analysis of NRF Transcriptional Targets

**Figure 3:**
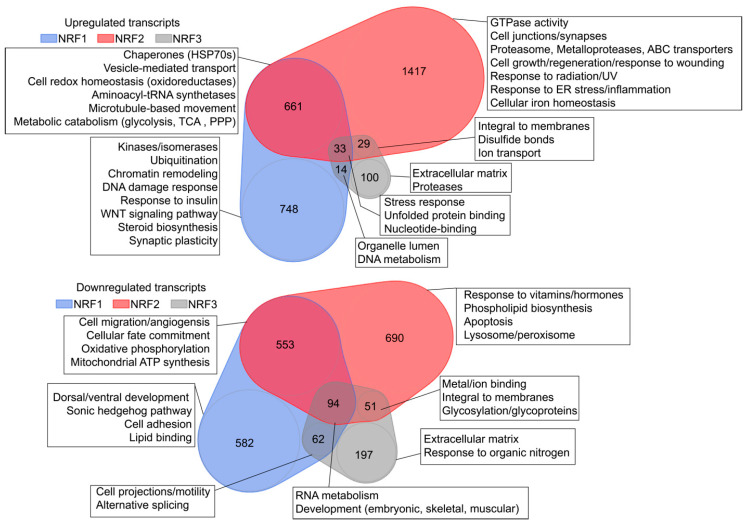
Venn diagrams depicting the number of transcripts upregulated (**top**) or downregulated (**bottom**) in response to overexpression of the indicated NRF transgenes. Boxes depict the results of pathway analyses of the indicated regions of Venn overlap.

HEK293T cells overexpressing NRF1, NRF2, or NRF3 were harvested 24 h post-transfection for RNA-seq analysis of transcript abundance using biological triplicates for each condition. Volcano and MA plots (Bland–Altman plot) demonstrated the robustness of our overexpression systems at manipulating the transcriptome of target cells (Supplementary Figures S1–S3; Supplementary Table S1), and DESeq-based analysis was used to identify differently expressed transcripts. Of 38,020 actively transcribed genes in HEK293T cells, 3002 transcripts were collectively upregulated and 2229 transcripts were collectively downregulated by NRF family overexpression relative to transiently transfected empty vector controls (*p* < 0.05). Specifically, we found that expression of NRF1 (1456 upregulated, 1291 downregulated) and NRF2 (2140 upregulated, 1388 downregulated) induced robust transcriptional programs in cells, comprising 7.2% and 9.3% of the active transcriptome, respectively. Interestingly, we observed considerably fewer transcripts were regulated by NRF3 overexpression (176 upregulated, 404 downregulated; 1.5% of active transcriptome), suggesting that NRF3 may enact a more muted transcriptional profile than NRF1 or NRF2 in HEK293T cells. From GO term-based analyses using DAVID, transcripts upregulated by all NRFs were found to be involved in stress responses, proteostasis-associated biological pathways, and nucleotide-binding, whereas downregulated transcripts were associated with RNA metabolism and development (Figure 3, top). As expected, transcripts upregulated by NRF2 are involved in transcriptional responses to radiation, ultraviolet exposure, endoplasmic reticulum stress, and inflammation, among other pathways associated with oxidative stress (Figure 3). NRF1-upregulated transcripts are associated with the processes of ubiquitination and chromatin remodeling and downregulated targets include those related to cell adhesion and the Sonic hedgehog pathway involved in embryonic development. NRF3-regulated transcripts are associated with the extracellular matrix, as well as an upregulation of transcripts related to the proteasome and downregulation of transcripts related to the cellular response to organic nitrogen. A comprehensive list of differentially regulated transcripts used for DAVID-based analysis of differential and overlapping targets can be found in Supplementary Table S2.

#### 3.2.2. Defining a Core Set of NRF2 Target Genes

**Figure 4:**
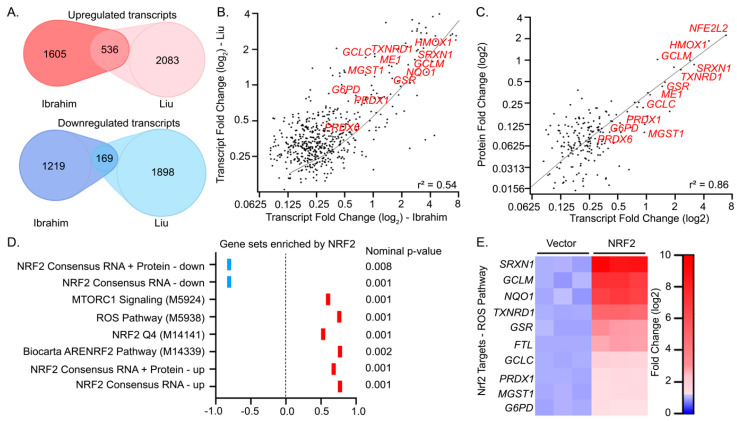
A consensus set of NRF2 target genes associated with oxidative stress resistance. (**A**) Venn diagram depicting the overlapping and differentially expressed transcripts induced by NRF2 activation identified in this work (Ibrahim, *p* < 0.05 and absolute value log_2_ fold change > 0.075) in comparison to those obtained with reported inducible U2OS-based system (Liu et al.). (**B**) Plot depicting the linear relationship of differentially expressed transcripts derived from the common targets in this work and from Liu et al. (**C**) Linear regression plot of log_2_ fold change values of protein and transcript levels of significantly changed genes in response to NRF2 overexpression. (**D**) Summary of GSEA analysis of RNA-seq profiling in response to NRF2 overexpression. (**E**) Heatmap depicting log_2_ fold change of transcript levels associated with a response to ROS that is upregulated by NRF2 overexpression by DAVID analysis.

Recently a report from Liu et al. used a tetracycline-inducible system to evaluate the transcriptional effects of NRF activation in U2OS cells [19]. As this work is the only other to comparatively profile the three NRF family members, we sought to generate consensus gene sets for the three NRF family members derived from our transcriptional profiling (hereafter referred to as “Ibrahim”) compared to the work of Liu et al. (hereafter referred to as “Liu”). We first characterized NRF2, as its expression profile has been best characterized in the literature and it induced the most robust transcriptional profile in cells. Approximately thirty percent of the differentially expressed transcripts we identified were corroborated by the Liu gene set (Figure 4A; Supplementary Table S3). A linear regression analysis comparing the mean log_2_ fold change of the RPKM values in cells overexpressing NRF2 relative to a vector control displayed a moderate correlation (r^2^ = 0.53) between differentially expressed transcripts identified by the Liu RNA-seq study and this work (Figure 4B). Among differentially expressed transcripts shared between Liu and Ibrahim, we observed a strong correlation of the log_2_ fold changes at the protein and transcript level (r^2^ = 0.86) (Figure 4C; Supplementary Table S4). This ultimately allowed us to generate a consensus gene set of 239 high confidence NRF2-regulated genes, which incorporates our proteomic and transcriptional profiling (Supplementary Table S5).

We next analyzed our RNA-seq data using GSEA to determine which transcriptional programs were most significantly modulated in response to NRF2 overexpression. For this analysis, we used a curated list of 6960 gene sets from the Molecular Signatures Database (MSigDB), a collection that spans many cellular pathways, treatment conditions, disease states, and cell types (Supplementary Table S6; Supplementary Figure S4). Of these gene sets, our data indicated that MTORC1 signaling (MSigDB: M5924), reactive oxygen species (ROS) pathways (MSigDB: M5938), NRF2 Q4 (MSigDB: M14141), and Biocarta ARENRF2 pathway (MSigDB: M14339) were most significantly enriched in cells overexpressing NRF2 (nominal *p*-value < 0.002; Figure 4D). In addition to the curated gene sets, we concurrently analyzed four gene sets derived from our transcriptomic and proteomic profiling. “NRF2 consensus RNA” genesets contain significantly upregulated or downregulated transcripts shared by Ibrahim and Liu, and the “NRF2 consensus RNA + Protein” gene set includes the NRF consensus RNA set but is restricted to genes whose protein levels are also significantly altered relative to vector controls. GSEA indicated these were among the most significantly up and downregulated sets evaluated relative to 6690 other gene sets used in this work (Figure 4D). Lastly, GO term (DAVID)-based analysis of the consensus upregulated gene sets indicated a strong association with elements of the ROS pathway (GO = 0006979; *p* < 0.01; Figure 4E; Supplementary Table S7). Together, this data agrees well with reported NRF2-controlled gene sets, suggesting our overexpression and profiling platform was suitable to evaluate the genetic targets of the less explored transcription factors NRF1 and NRF3.

#### 3.2.3. Defining a Core Set of NRF1 Target Genes

**Figure 5:**
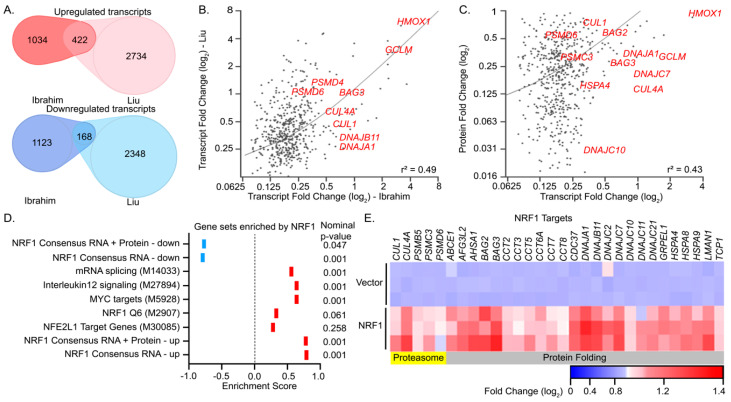
A consensus set of NRF1 target genes associated with proteostasis. (**A**) Venn diagram depicting the overlapping and differentially expressed transcripts induced by NRF1 activation identified in this work (Ibrahim, *p* < 0.05 and absolute value of log_2_ fold change > 0.075) in comparison to those obtained with reported inducible U2OS-based system (Liu et al.). (**B**) Plot depicting the linear relationship of differentially expressed transcripts derived from the common NRF1 targets in this work and from Liu et al. (**C**) Linear regression plot of log_2_ fold change values of protein and transcript levels of significantly altered genes in response to NRF1 overexpression. (**D**) Summary of GSEA analysis of RNA-seq profiling in response to NRF1 overexpression (**E**) Heatmap depicting log_2_ fold change of transcript levels associated with proteostasis that are upregulated by NRF2 overexpression by DAVID analysis.

We next analyzed the resulting transcriptomes and proteomes of HEK293T cells overexpressing NRF1 using the analysis pipeline described above. Approximately twenty-two percent of the differentially expressed transcripts identified by our RNA-seq analysis of cells overexpressing NRF1 were corroborated by the Liu set (Figure 5A). Linear regression analysis comparing the mean log_2_ fold change of the RPKM values in cells overexpressing NRF1 relative to vector controls demonstrated a moderate correlation (r^2^ = 0.49) between the Liu study and ours (Figure 5B). Likewise, these differentially controlled transcripts common to the Liu study, and this work was found to be correlated with the log_2_ fold changes at the protein level (r^2^ = 0.49) (Figure 5C). These analyses suggest a high confidence set of 248 genetic targets regulated by NRF1.

GSEA of our RNA-sequencing dataset indicated that the most significantly upregulated gene sets associated with NRF1 overexpression corresponded to mRNA splicing (MSigDB: M14033, ES = 0.6), IL12 Signaling (MSigDB: M27894, ES = 0.6) and MYC targets (MSigDB: M5928, ES = 0.6; Supplementary Figure S5). Unlike NRF2, there are only two potential NRF1-related genesets in the MSigDB, NRF1 Q6 (MSigDB: M2907) and NFE2L1 Target Genes ID (MSigDB: M30085), which correspond to genes containing predicted NRF1 binding sites in their genetic loci and do not indicate if the transcription is up or downregulated in response to NRF1 activation [16,17]. Accordingly, these sets were not significantly enriched by NRF1 overexpression in this work (Figure 5D). GO term analysis of our high confidence set of 236 genes upregulated by NRF1 indicated that in addition to the proteasome-related genes (e.g., *PSMB5*, *PSMC3*, *PSMD6*), NRF1 activation additionally promotes the upregulation of gene products involved in protein folding, including multiple classes of chaperones (GO = 0051082; *p* < 0.005; Figure 5E; Supplementary Table S8). Although it is unclear if these chaperone genes are direct targets of NRF1, the consensus identification of chaperones in the Liu and Ibrahim RNA-seq analyses indicate a functional link between NRF1 activity and adaptive remodeling of cellular proteostasis in response to cellular stress.

#### 3.2.4. Defining a Core Set of NRF3 Target Genes

**Figure 6:**
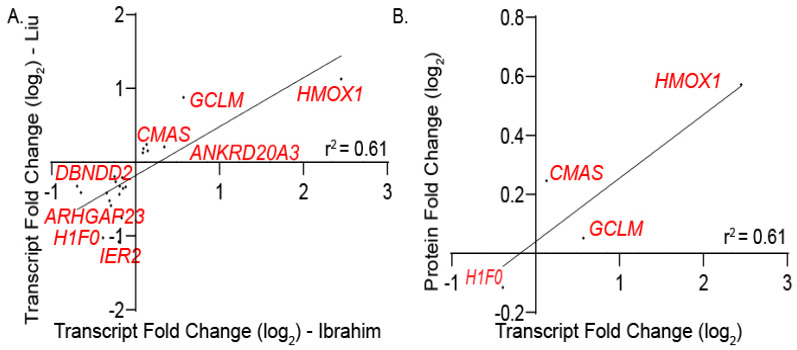
Defining common NRF3 targets within transcriptomes and proteomes. (**A**) Plot depicting the linear relationship of differentially expressed transcripts derived from the common NRF3 targets in this work and from Liu et al. (**B**) Linear regression plot of log_2_ fold change values of protein and transcript levels of significantly modulated genes in response to NRF1 overexpression.

Applying the same RNA-seq analysis pipeline to samples overexpressing NRF3, 23 differentially regulated transcripts were found to be shared between the Liu dataset and those found in this work (Figure 6A). Of these, four gene products (HMOX1, CMAS, GCLM, and F1F0) were additionally found by our mass spectrometry-based analysis to be regulated at the protein level by NRF3 overexpression. (Figure 6B). This consensus set of four proteins was insufficient to gain meaningful GO term analysis using DAVID, limiting our ability to define a clear NRF3 gene set through this analysis (Supplementary Figure S6).

### 3.3. Identifying the Co-Expression Patterns of NRF Target Genes across Human Tissues

The gene sets defined herein, especially those for NRF1 and NRF2, provide new opportunities to define selective activation of these transcription factors in cellular models. However, we sought to expand this study to determine the integrity of these gene sets for defining activation of these transcriptional programs in human tissues. A challenge in applying this gene set-based approach to in vivo models is the potential for differential regulation of these pathways in different tissues. For example, core NRF2 target genes identified in our study may be similarly regulated in some tissues but not others. Further, genes identified as targets of NRF transcription factors herein could demonstrate tissue-specific regulation by other transcription factors that reduce their fidelity for selectively reporting on NRF activation. One way to address these potential challenges is to identify subsets of the genesets defined herein that report on NRF transcription factor activity in different tissues.

**Figure 7:**
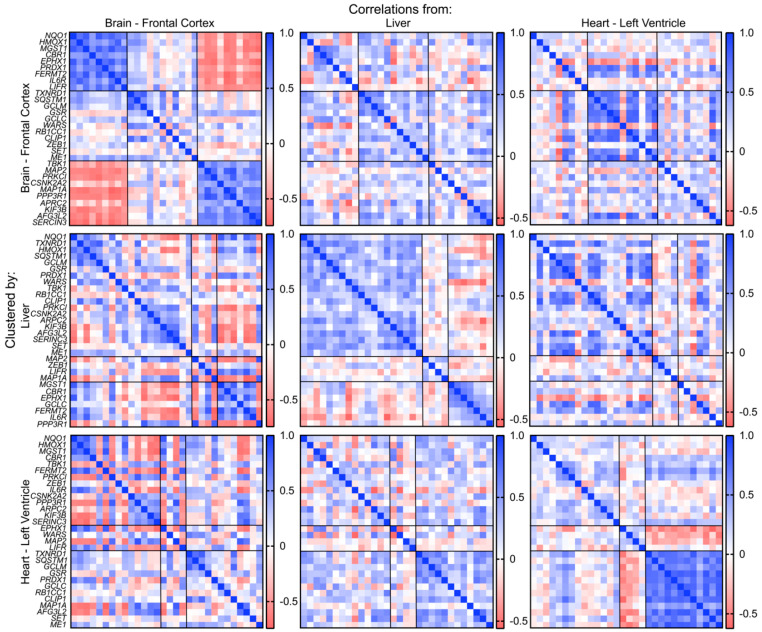
Co-expression patterns of NRF2 target transcripts in human tissues. Genes are separated into three groups by hierarchical clustering. First row: Correlation matrices of brain, liver, and heart clustered by the similarity of correlation patterns in the brain. Second row: Correlation matrices of brain, liver, and heart clustered by the similarity of correlation patterns in the liver. Third row: Correlation matrices of brain, liver, and heart clustered by the similarity of correlation patterns in the heart.

Recently, a collaborative effort to comprehensively profile the diverse transcriptional programs enacted across tissues has led to the inception of the Genotype-Tissue Expression Project (GTEx). GTEx catalogs the RNA-sequencing-based quantification of transcript levels from 54 postmortem tissues derived from over 1000 individuals, providing a unique opportunity to probe the coordinated regulation of specific genes across multiple tissues. Given the comprehensiveness of this dataset, we leveraged the GTEx database to determine if the high confidence, consensus gene sets generated for NRF transcription factors were co-regulated across tissues using correlative analyses for co-expression. Correlation coefficients were calculated between the expression levels of each pair of genes in the NRF1, NRF2, and NRF3 gene sets in the frontal cortex of the brain, the liver, and the left ventricle of the heart. The gene sets for each transcription factor were then clustered in each tissue into three groups using k-means clustering based on the Euclidean distance between each gene’s correlation coefficients. Surprisingly, among the thirty genes categorized as upregulated by NRF2 overexpression, we observed significant differences in gene co-expression across tissues (Figure 7; Table 1). For example, while canonical NRF2 target genes including *NQO1*, *HMOX1*, and *MST1* showed strong co-expression correlation across the three tissues, other canonical target genes such as *GCLM* and *GCLC* showed variable correlation across the tissues, indicating that there is tissue-specific variability in co-expression relationships for consensus NRF2 gene targets. Similar variability in co-expression was observed for both NRF1 and NRF3 gene sets (Supplementary Figures S7 and S8; Supplementary Table S9). These results suggest that while the NRF gene sets defined herein are suitable for probing selective activation of these pathways induced by genetic or pharmacologic approaches, in complex physiologic settings of human tissues the contributions of other transcription factors and/or the overlapping nature of these gene sets challenge the ability to define the activation of these pathways using this approach.

## 4. Discussion

Here, we have used a transient overexpression system to define the functional targets of the NRF transcription factors NRF1, NRF2, and NRF3. Our work serves as a complement to the study of Liu et al., which aimed to profile the transcriptional targets of the NRF family. Here we have used both transcriptomics and quantitative proteomics to define how NRF1, NRF2, and NRF3 sculpt the proteomes and transcriptomes of mammalian cells. Collectively, we defined genetic targets corresponding to overlapping and distinct transcriptional targets among NRF transcription factors and used an analysis pipeline which incorporates transcriptional profiling, proteomics, and the data of Liu et al. to define high confidence target lists for NRF1, NRF2, and to a lesser extent NRF3. The genetic programs described herein will likely be of key utility in further defining the role of the NRF transcription factors.

One of the most salient observations from our profiling work involved the composition of the high confidence NRF1 target gene set. Included in this repertoire of NRF1 targets were a number of chaperones including heat shock proteins (e.g., HSPA4, HSPA8, HSPA9, DNAJC1, DNAJA2) and the chaperonin TCP complex (e.g., CCT2, CCT5, CCT8), a gene set which corresponded with a heat shock response by GO analysis. Previously, NRF1 has been described as mediating the proteasomal ‘bounce back’ response to proteasome inhibitor treatment by augmenting the transcription of components of the 20S proteasome and 19S regulatory complex, but a role in mediating the transcription of chaperones has not been described [11]. Our work suggests that NRF1 may also play a role in cellular protein quality control, not only by increasing the degradation of misfolded proteins through increased proteasomal numbers but also by upregulating genes products actively involved in protein folding. Interestingly, brain-specific knockout of NRF1 in mice results in the age-dependent increase in aggregated proteins, brain atrophy, and decreased motor capacity, as is observed in neurodegenerative disease [30]. We and others have reported the discovery of non-toxic small molecule activators of NRF1 activity in cells, pharmacological tools which will likely be of utility in understanding if the NRF1 transcriptional program might be augmented for proteostasis-based repair in disease [26].

Surprisingly, our transcriptional profiling results suggested that NRF3 enacted a considerably smaller transcriptional program relative to NRF1 or NRF2. This observation as well as the moderate overlap in NRF3 targets between this work and the work of Liu et al. may indicate that NRF3 activates a tissue-specific transcriptional program or requires additional yet undescribed co-regulatory machinery for robust transcriptional activation. Interestingly NRF3 is not broadly expressed across tissues like NRF1 and NRF2. Instead, NRF3 displays uneven tissue distribution, with the highest expression in the placenta and other female-specific tissues. Ultimately, further work understanding the upstream stimuli which promote NRF3 activation and delineating in which cell types and in what physiological contexts NRF3 is active will help uncover the role of this cryptic transcription factor.

As this work represents the first time that consensus genetic targets derived from multiple studies could be defined for the NRF family members, we sought to understand how the consensus sets generated in this work might be co-regulated across human tissues. In the simplest circumstance, one might expect the expression levels of genes within each set to be a function of the active levels of a given NRF member in that tissue, which would result in high correlation coefficients among all or nearly all genes within each gene set. Unexpectedly, we found that most NRF1, NRF2, and NRF3 core consensus gene sets were not co-regulated basally in tissues but instead displayed tissue specific preferences in their expression, as co-regulated genes within a set within one organ were often not correlated within another organ. This observation likely spells out the existence of other transcription factors or epigenetic states playing a predominant role in regulating these genes basally in tissue. Often stress-responsive transcriptional programs are treated collectively, as investigators frequently use one or two representative transcripts to report on the entirety of a transcriptional program. This data suggests caution in pursuing such an approach, as our data suggest that even genes perceived to be core transcriptional targets within a given set are not basally co-regulated. Thus, we believe this result to be of critical importance in studying the regulation of stress-responsive signaling in tissue. Instead, our data provide a preliminary roadmap for evaluating the expression and the relationships of NRF-driven transcripts in tissue.

## 5. Conclusions

Here, we have used a transient overexpression system in conjunction with RNA-seq and proteomic profiling to annotate the functional genetic programs enacted by the Cap‘n’collar transcription factors NRF1, NRF2, and NRF3. Integrating the work of a complementary study performed by Liu et al., we have defined the consensus genetic targets of the NRF family members from experiments performed with human cell lines. Analysis of the comprehensive tissue expression GTEx indicated these gene sets are likely not basally co-regulated in human tissues but instead display organ-specific co-expression patterns. Together, our data provide a useful resource for future lines of inquiry aimed at understanding the roles of NRF transcription factors in normal physiology and disease. 

## Figures and Tables

**Table 1:** Correlated and anti-correlated NRF2 target genes in the indicated human tissues. Related to Figure 7.

Brain		Liver		Heart	
Correlated	Anti-Correlated	Correlated	Anti-Correlated	Correlated	Anti-Correlated
*NQO1*	*TBK1*	*NQO1*	*MGST1*	*NQO1*	*TXNRD1*
*HMOX1*	*MAP2*	*TXNRD1*	*CBR1*	*HMOX1*	*SQSTM1*
*MGST1*	*PRKCI*	*HMOX1*	*EPHX1*	*MGST1*	*GCLM*
*CBR1*	*CSNK2A2*	*SQSTM1*	*GCLC*	*CBR1*	*GSR*
*EPHX1*	*MAP1A*	*GCLM*	*FERMT2*	*TBK1*	*PRDX1*
*PRDX1*	*PPP3R1*	*GSR*	*IL6R*	*FERMT2*	*GCLC*
*FERMT2*	*ARPC2*	*PRDX1*	*PPP3R1*	*PRKCI*	*RB1CC1*
*IL6R*	*KIF3B*	*WARS*		*ZEB1*	*CLIP1*
*LIFR*	*AFG3L2*	*TBK1*		*IL6R*	*MAP1A*
	*SERINC3*	*RB1CC1*		*CSNK2A2*	*AFG3L2*
		*CLIP1*		*PPP3R1*	*SET*
		*PRKCI*		*ARPC2*	*ME1*
		*CSNK2A2*		*KIF3B*	
		*ARPC2*		*SERINC3*	
		*KIF3B*			
		*AFG3L2*			
		*SERINC3*			
		*SET*			
		*ME1*			

## Supplementary Materials

The following are available online at https://www.mdpi.com/2076-3921/9/10/1025/s1, Figure S1: Overview of transcriptomic and proteomic profiling of HEK293T cells overexpressing NRF1, Figure S2: Overview of transcriptomic and proteomic profiling of HEK293T cells overexpressing NRF2, Figure S3: Overview of transcriptomic and proteomic profiling of HEK293T cells overexpressing NRF3, Figure S4: GSEA analysis of HEK293T cells overexpressing NRF1, Figure S5: GSEA analysis of HEK293T cells overexpressing NRF2, Figure S6: GSEA analysis of HEK293T cells overexpressing NRF3, Figure S7: Co-expression patterns of NRF1 target transcripts in human tissues, Figure S8: Co-expression patterns of NRF3 target transcripts in human tissues, Table S1: RPKM values for transcripts identified by RNA-seq, Table S2: Differentially expressed transcripts identified by RNA-seq analysis, Table S3: Consensus differentially expressed transcripts between Ibrahim and Liu RNA-seq analyses, Table S4: Fold changes of m/z values for proteins identified by mass spectrometry between NRF samples and empty vector controls, Table S5: High confidence NRF2-regulated genes incorporating transcriptomic and proteomic profiling, Table S6: Curated list of gene sets from the Molecular Signatures Database (MSigDB), including gene sets derived from our transcriptomic and proteomic profiling, Table S7: DAVID analysis of high confidence targets of NRF2, Table S8: DAVID analysis of high confidence targets of NRF1, Table S9: Correlated and anti-correlated NRF1 and NRF3 target genes in human tissues. Related to Supplementary Figures S7 and S8.
