# Supplementary material for: Defining the Functional Targets of Cap‘n’collar Transcription Factors NRF1, NRF2, and NRF3
Source: Antioxidants (Basel). 2020 Oct 21;9(10):1025. doi: 10.3390/antiox9101025 (PMC7588902; doi:10.3390/antiox9101025)
Supplement: Supplementary file 1 [file antioxidants-09-01025-s001.zip › antioxidants-955093-suppl/antioxidants-955093-suppl.pdf]

Supplementary Materials to the Manuscript:

Defining the Functional Targets of Cap'n'collar Transcription Factors NRF1, NRF2, and NRF3.

Lara Ibrahim, Jaleh Mesgarzadeh, Ian Xu, Evan T. Powers, R. Luke Wiseman and Michael J. Bollong

Correspondence: mbollong@scripps.edu

The supplementary material comprises eight figures and nine tables:

Figure S1: Overview of transcriptomic and proteomic profiling of HEK293T cells overexpressing NRF1, Figure S2: Overview of transcriptomic and proteomic profiling of HEK293T cells overexpressing NRF2, Figure S3: Overview of transcriptomic and proteomic profiling of HEK293T cells overexpressing NRF3, Figure S4: GSEA analysis of HEK293T cells overexpressing NRF1, Figure S5: GSEA analysis of HEK293T cells overexpressing NRF2, Figure S6: GSEA analysis of HEK293T cells overexpressing NRF3, Figure S7: Co-expression patterns of NRF1 target transcripts in human tissues, Figure S8: Co-expression patterns of NRF3 target transcripts in human tissues, Table S1: RPKM values for transcripts identified by RNA-seq, Table S2: Differentially expressed transcripts identified by RNA-seq analysis, Table S3: Consensus differentially expressed transcripts between Ibrahim and Liu RNA-seq analyses, Table S4: Fold changes of m/z values for proteins identified by mass spectrometry between NRF samples and empty vector controls, Table S5: High confidence NRF2-regulated genes incorporating transcriptomic and proteomic profiling, Table S6: Curated list of gene sets from the Molecular Signatures Database (MSigDB), including gene sets derived from our transcriptomic and proteomic profiling, Table S7: DAVID analysis of high confidence targets of NRF2, Table S8: DAVID analysis of high confidence targets of NRF1, Table S9: Correlated and anti-correlated NRF1 and NRF3 target genes in human tissues. Related to Supplementary Figures 7 and 8.

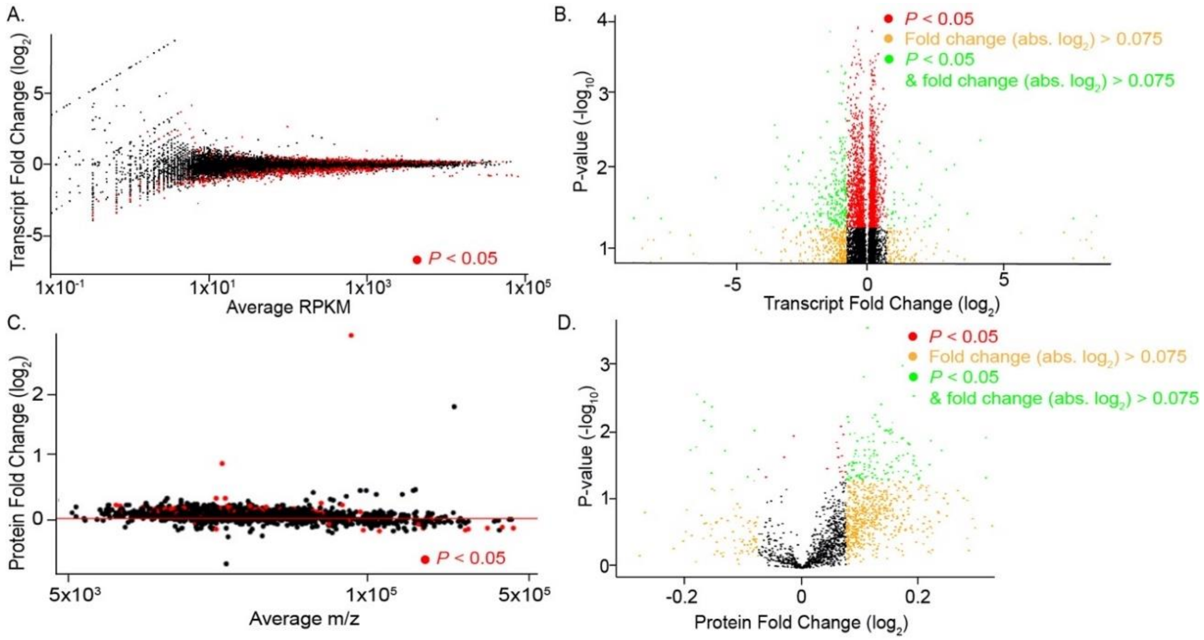

Supplementary Figure 1. Overview of transcriptomic and proteomic profiling of HEK293T cells overexpressing NRF1. (**A.**) MA plot representing transcripts identified by RNA-sequencing. Points in red represent transcripts with a statistically significant fold change (NRF1 vs. vector; $P < 0.05$). (**B.**) Volcano plot representing transcripts identified by RNA-sequencing. Points in red represent transcripts with a fold change (NRF1 vs. vector; $P < 0.05$). Points in orange represent transcripts with a fold change greater than 0.05 (absolute value of log_2). Green points satisfy both conditions. (**C.**) MA plot representing proteins identified by mass spectrometry. Points in red represent proteins with a statistically significant fold change (NRF1 vs. vector; $P < 0.05$). (**D.**) Volcano plot representing proteins identified by mass spectrometry. Points in red represent proteins with a statistically significant fold change (NRF1 vs. vector; $P < 0.05$). Points in orange represent proteins with a fold change > 0.05 (absolute value of log_2). Green points satisfy both conditions.

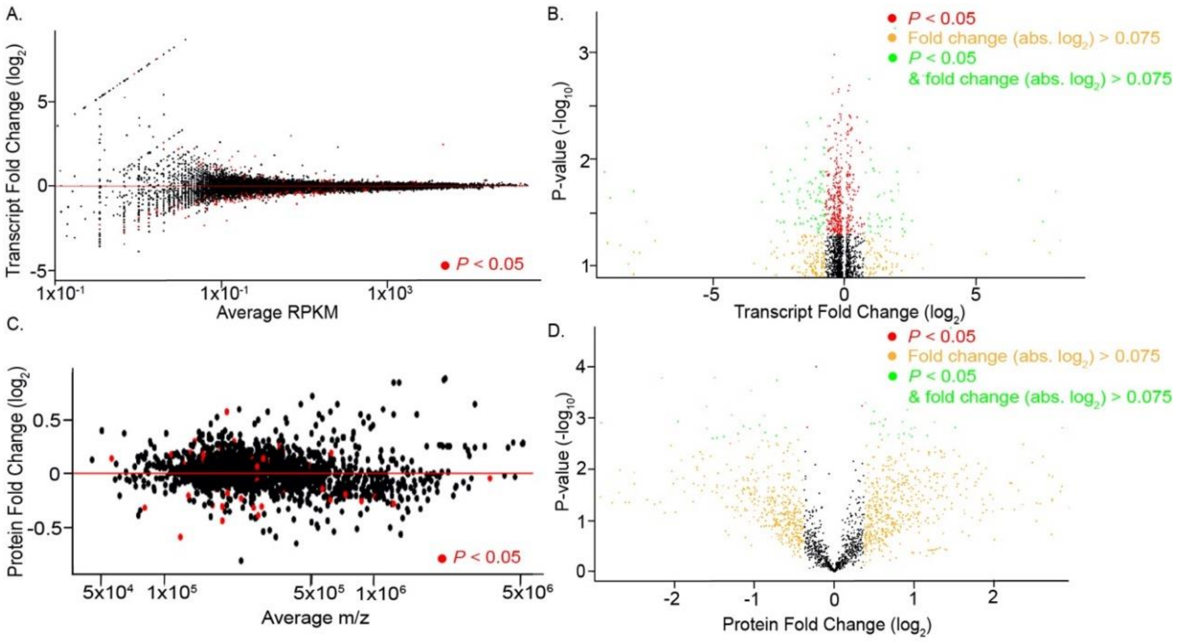

Supplementary Figure 2. Overview of transcriptomic and proteomic profiling of HEK293T cells overexpressing NRF2. (**A.**) MA plot representing transcripts identified by RNA-sequencing. Points in red represent transcripts with a statistically significant fold change (NRF2 vs. vector; $P < 0.05$). (**B.**) Volcano plot representing transcripts identified by RNA-sequencing. Points in red represent transcripts with a fold change (NRF2 vs. vector; $P < 0.05$). Points in orange represent transcripts with a fold change greater than 0.05 (absolute value of $\log_2$). Green points satisfy both conditions. (**C.**) MA plot representing proteins identified by mass spectrometry. Points in red represent proteins with a statistically significant fold change (NRF2 vs. vector; $P < 0.05$). (**D.**) Volcano plot representing proteins identified by mass spectrometry. Points in red represent proteins with a statistically significant fold change (NRF2 vs. vector; $P < 0.05$). Points in orange represent proteins with a fold change > 0.05 (absolute value of $\log_2$). Green points satisfy both conditions.

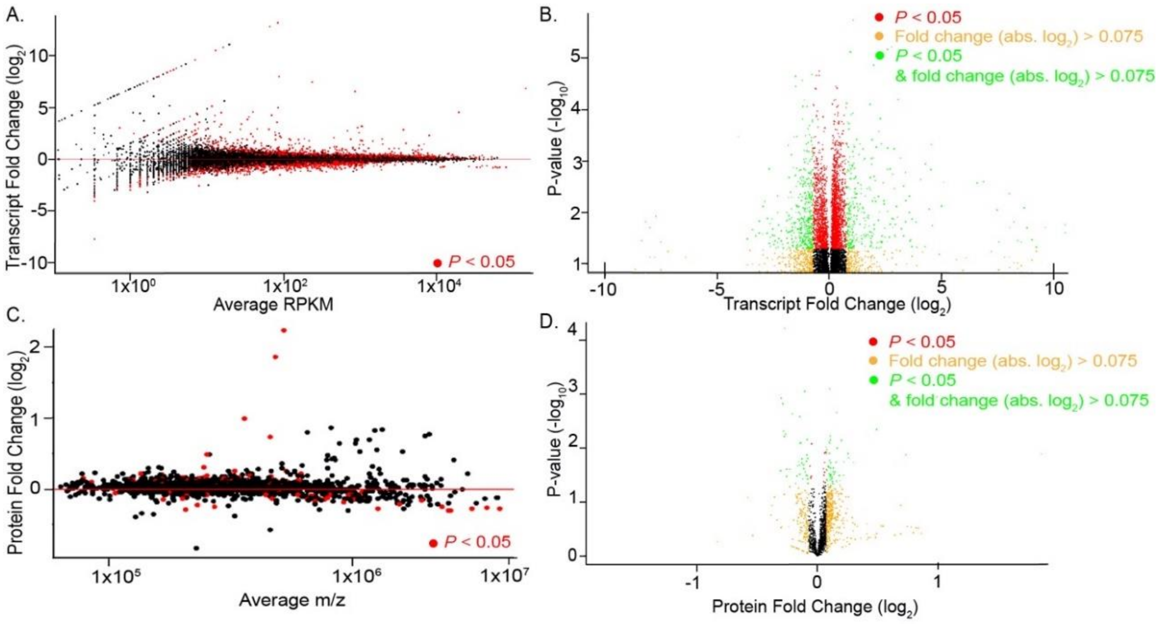

Supplementary Figure 3. Overview of transcriptomic and proteomic profiling of HEK293T cells overexpressing NRF3. (**A.**) MA plot representing transcripts identified by RNA-sequencing. Points in red represent transcripts with a statistically significant fold change (NRF3 vs. vector; $P < 0.05$). (**B.**) Volcano plot representing transcripts identified by RNA-sequencing. Points in red represent transcripts with a fold change (NRF3 vs. vector; $P < 0.05$). Points in orange represent transcripts with a fold change greater than 0.05 (absolute value of $\log_2$). Green points satisfy both conditions. (**C.**) MA plot representing proteins identified by mass spectrometry. Points in red represent proteins with a statistically significant fold change (NRF3 vs. vector; $P < 0.05$). (**D.**) Volcano plot representing proteins identified by mass spectrometry. Points in red represent proteins with a statistically significant fold change (NRF3 vs. vector; $P < 0.05$). Points in orange represent proteins with a fold change > 0.05 (absolute value of $\log_2$). Green points satisfy both conditions.

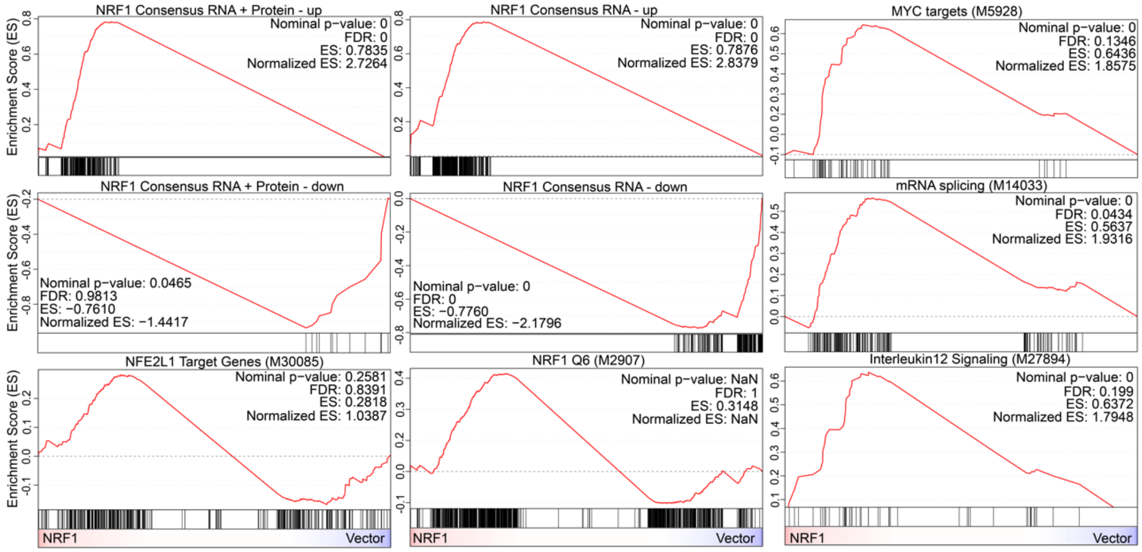

Supplementary Figure 4. GSEA analysis of HEK293T cells overexpressing NRF1.

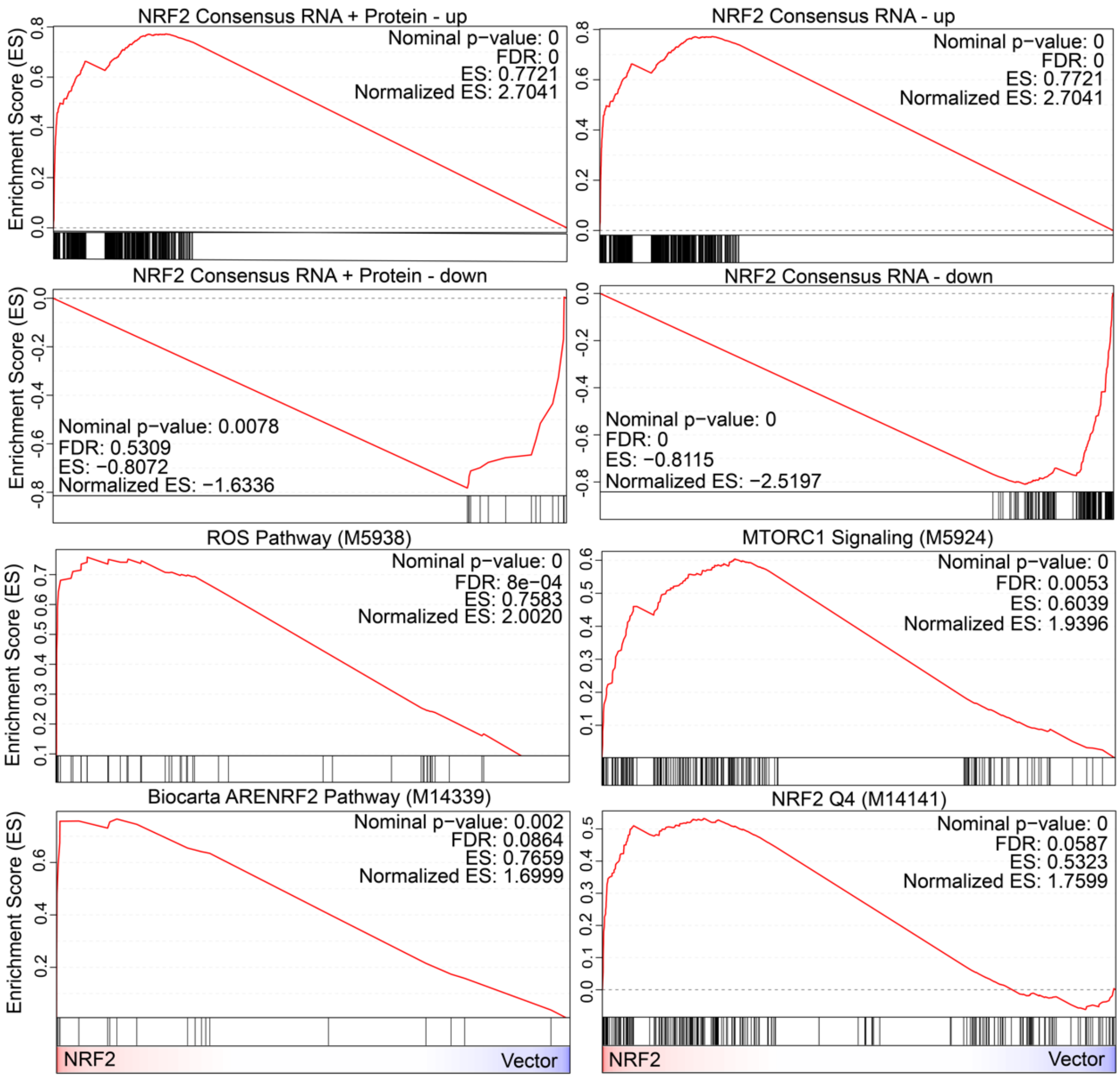

Supplementary Figure 5. GSEA analysis of HEK293T cells overexpressing NRF2.

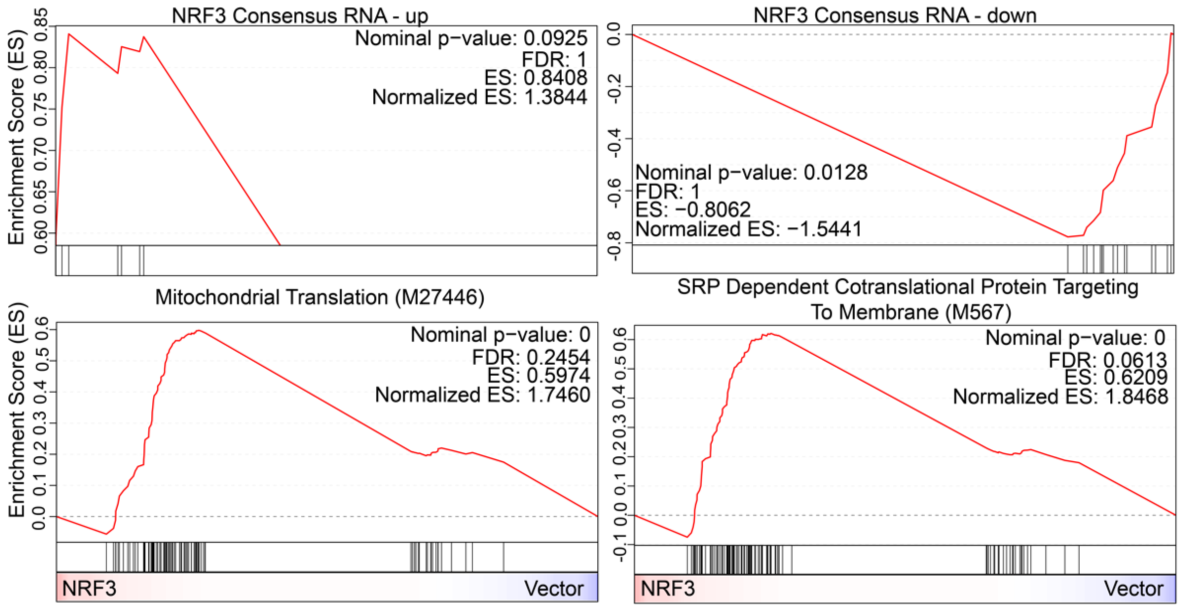

Supplementary Figure 6. GSEA analysis of HEK293T cells overexpressing NRF3.

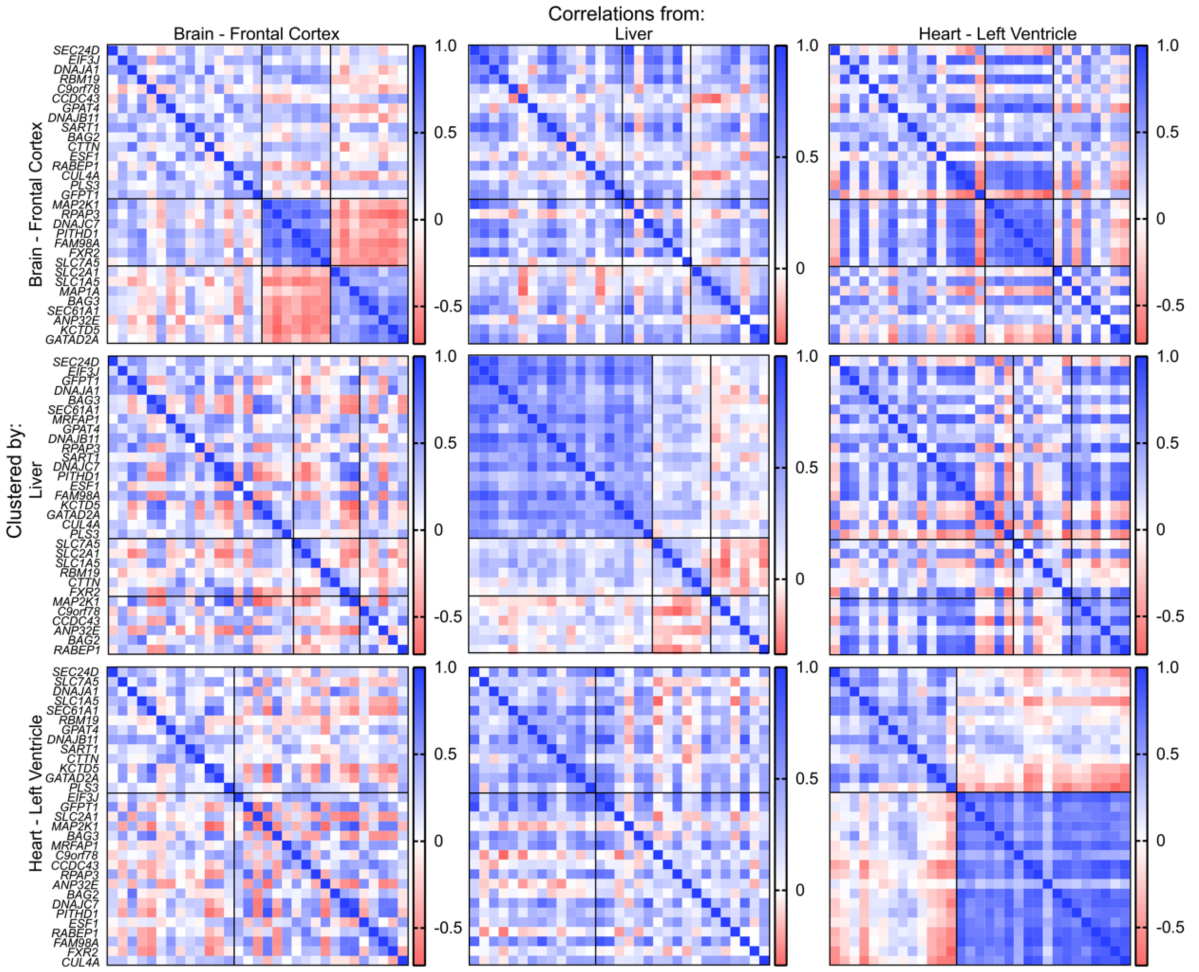

Supplementary Figure 7. Co-expression patterns of NRF1 target transcripts in human tissues. Genes are separated into three groups by hierarchical clustering. First row: Correlation matrices of brain, liver, and heart clustered by similarity of correlation patterns in the brain. Second row: Correlation matrices of brain, liver, and heart clustered by similarity of correlation patterns in the liver. Third row: Correlation matrices of brain, liver, and heart clustered by similarity of correlation patterns in the heart.

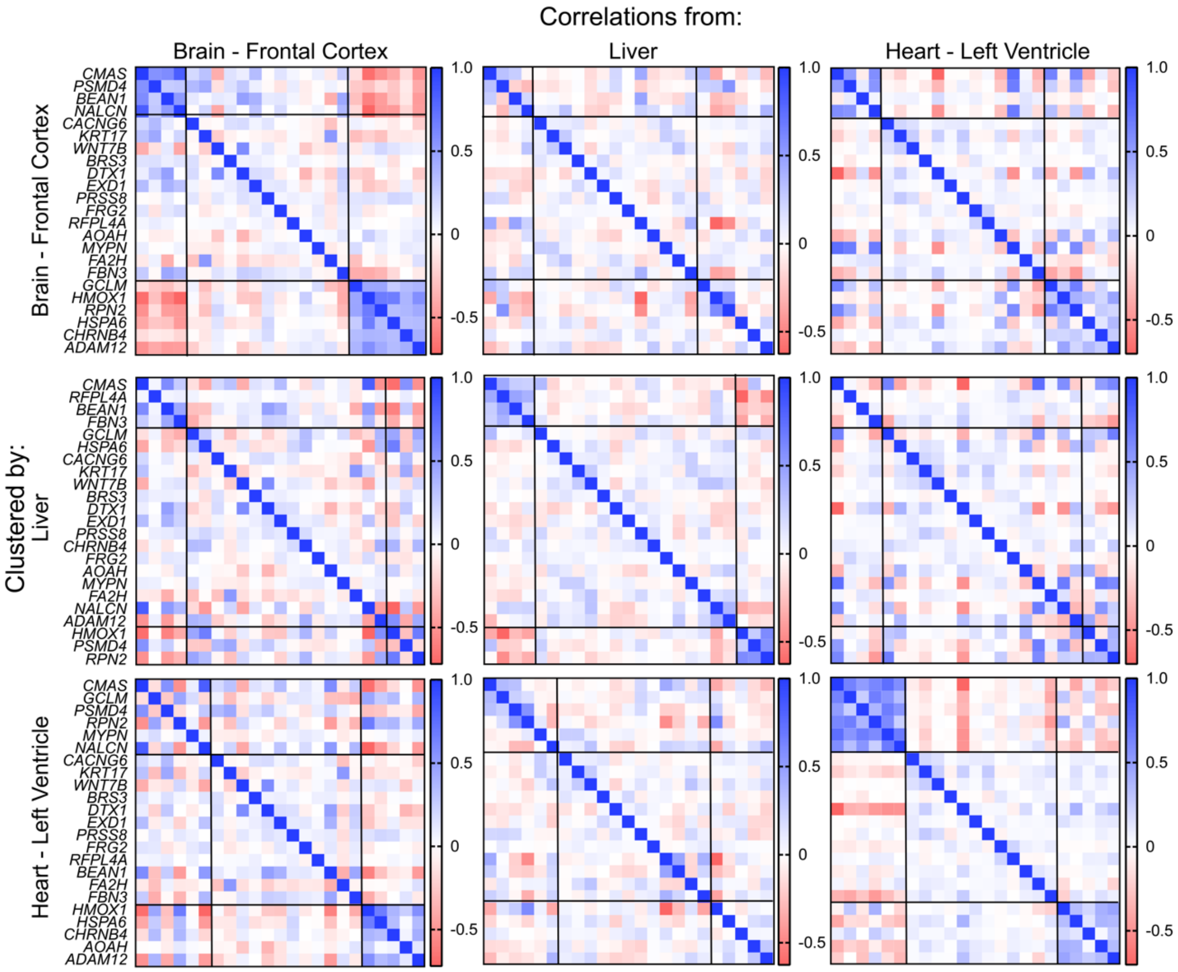

Supplementary Figure 8. Co-expression patterns of NRF3 target transcripts in human tissues. Genes are separated into three groups by hierarchical clustering. First row: Correlation matrices of brain, liver, and heart clustered by similarity of correlation patterns in the brain. Second row: Correlation matrices of brain, liver, and heart clustered by similarity of correlation patterns in the liver. Third row: Correlation matrices of brain, liver, and heart clustered by similarity of correlation patterns in the heart.
